# Identification of Anti-Malarial Compounds as Novel Antagonists to Chemokine Receptor CXCR4 in Pancreatic Cancer Cells

**DOI:** 10.1371/journal.pone.0031004

**Published:** 2012-02-03

**Authors:** Joseph Kim, M. L. Richard Yip, Xiaoming Shen, Hubert Li, Li-Yu Charlie Hsin, Samuel Labarge, Eileen L. Heinrich, Wendy Lee, Jianming Lu, Nagarajan Vaidehi

**Affiliations:** 1 Department of Surgery, City of Hope, Duarte, California, United States of America; 2 Department of Molecular Medicine, City of Hope, Duarte, California, United States of America; 3 Department of Immunology, City of Hope, Duarte, California, United States of America; Indiana University School of Medicine, United States of America

## Abstract

Despite recent advances in targeted therapies, patients with pancreatic adenocarcinoma continue to have poor survival highlighting the urgency to identify novel therapeutic targets. Our previous investigations have implicated chemokine receptor CXCR4 and its selective ligand CXCL12 in the pathogenesis and progression of pancreatic intraepithelial neoplasia and invasive pancreatic cancer; hence, CXCR4 is a promising target for suppression of pancreatic cancer growth. Here, we combined *in silico* structural modeling of CXCR4 to screen for candidate anti-CXCR4 compounds with *in vitro* cell line assays and identified NSC56612 from the National Cancer Institute's (NCI) Open Chemical Repository Collection as an inhibitor of activated CXCR4. Next, we identified that NSC56612 is structurally similar to the established anti-malarial drugs chloroquine and hydroxychloroquine. We evaluated these compounds in pancreatic cancer cells *in vitro* and observed specific antagonism of CXCR4-mediated signaling and cell proliferation. Recent *in vivo* therapeutic applications of chloroquine in pancreatic cancer mouse models have demonstrated decreased tumor growth and improved survival. Our results thus provide a molecular target and basis for further evaluation of chloroquine and hydroxychloroquine in pancreatic cancer. Historically safe in humans, chloroquine and hydroxychloroquine appear to be promising agents to safely and effectively target CXCR4 in patients with pancreatic cancer.

## Introduction

Pancreatic duct cancer is a uniformly fatal disease that is frequently diagnosed with distant metastasis at the time of initial clinical presentation. Unrecognized early disease and a highly invasive phenotype are primary factors for the poor prognosis associated with pancreatic cancer and highlight the urgency to identify molecular targets for the progression of the disease. Recently, the interactions between chemokines and their corresponding receptors have been examined in the pathogenesis, progression, and metastasis of pancreatic cancer [1], [2], [3]. These studies have suggested that antagonists to chemokine receptor CXCR4 may abrogate the invasive phenotype of pancreatic cancer [4], [5], [6]. Despite increasing evidence to the importance of CXCR4 in pancreatic cancer and other malignancies, antagonists to CXCR4 that are safe and effective for clinical use remain lacking.

Chemokine CXCL12 (also known as stromal-derived factor-1α, SDF-1α) activates multiple downstream effector pathways upon binding its receptor CXCR4 [7]. The CXCL12-CXCR4 interaction regulates chemotaxis, adhesion, and secretion of growth factors among many of its known functions [8]. Shortly after CXCR4 was identified as a co-receptor for HIV-1 and HIV-2 [9], [10], the small bicyclam molecule AMD3100 was identified as a specific CXCR4 antagonist [5]. AMD3100 has now been widely used to investigate and interrogate CXCL12-CXCR4 interactions [7]. Although AMD3100 remains in clinical use for stem cell mobilization, its chronic administration has been associated with significant cardiotoxicity [11]. Interestingly, recent studies have shown that in addition to its role as an antagonist to CXCR4 signaling, AMD3100 paradoxically binds and activates chemokine receptor CXCR7 [12], [13].

Since current data suggests that AMD3100 may not be safe or effective as an anti-CXCR4 antagonist for therapeutic applications in pancreatic cancer, specific antagonists remain to be identified for this purpose. In this interdisciplinary investigation, we combined *in silico* modeling of CXCR4 structure with high-throughput screening and *in vitro* assays in pancreatic cancer cell lines to identify novel antagonists to CXCR4-mediated cell proliferation in pancreatic cancer cells. Our study shows that the safe and efficacious anti-malarial drugs chloroquine and hydroxychloroquine are effective CXCR4 antagonists that suppress pancreatic cancer cell proliferation.

## Results

### Computational Modeling of CXCR4

**Figure 1:**
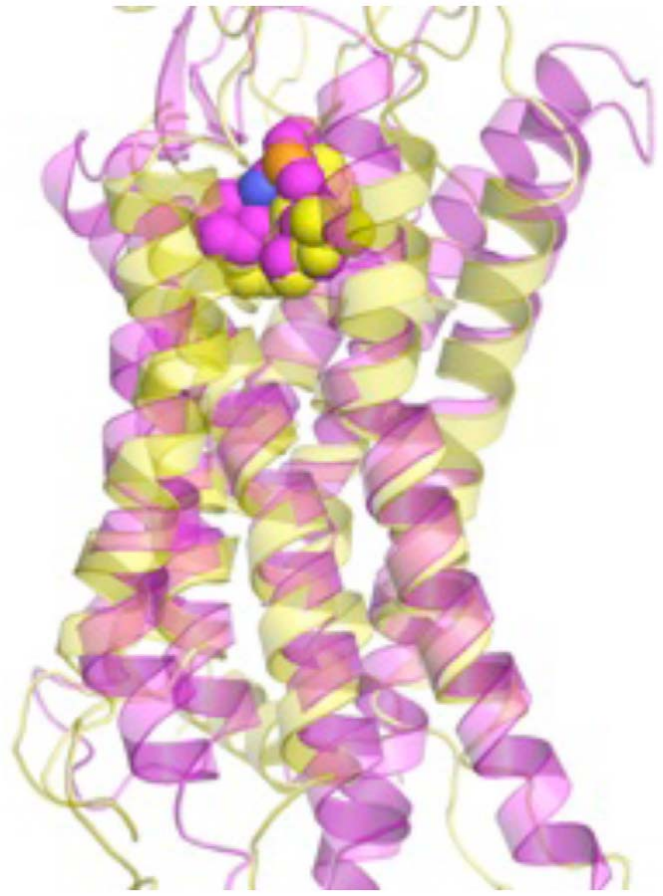
Comparison of the predicted structural model of CXCR4 (yellow) with the crystal structure (pink). The small molecule designated “1t” is placed into the predicted binding site. The root mean square deviation of the predicted and crystal structures is 2.5 Å, which demonstrates close alignment of our predicted model with the established crystal structure. Accordingly, the predicted location of the binding site of the small molecule “1t” matched with the crystal structure. The small molecule “1t” is depicted as small spheres.

The structural ensemble of the wild-type CXCR4 receptor was predicted using the *ab initio* structure prediction method (MembStruk4.3) [14], [15]. We compared the binding of mono and bicyclam compounds to our predicted structures with mutagenesis data to validate our computational predictions [16]. Our predictions were submitted to the protein structure assessment competition (GPCRDOCK2010) prior to the characterization of the crystal structure of CXCR4 [17]. A detailed comparison of the predicted structure with the crystal structure has verified the accuracy of our modeling and has been published elsewhere (Figure 1) [18].

We performed virtual ligand screening (VLS) of the National Cancer Institute's (NCI) Open Chemical Repository Collection for 3 different predicted conformations of CXCR4. Next, the candidate small molecules were filtered based on their proximity to residues that play an important role in antagonist binding, namely: D92 (TM2), H121 (TM3), D171 (TM4), E262 (TM6) and E288 (TM7) [19], [20]. Approximately 90% of the small molecules were excluded at this step.

Binding energies of the small molecules were then calculated and the top 10% of the small molecules with the lowest binding energies were retained. The chemical structures in the top 10% of the hits ranged from multi-aromatic ring structures to structures with longer alkyl chains. The primary criterion for further selection was the interaction of the candidate molecules with the residues that are known to be important for antagonist binding [16]. These molecules were then examined for protein-ligand contacts and 50 candidate small molecules were selected from approximately 350,000 molecules for experimental testing.

### NSC56612 and Related Compounds Inhibit CXCR4-Mediated Signaling

**Figure 2:**
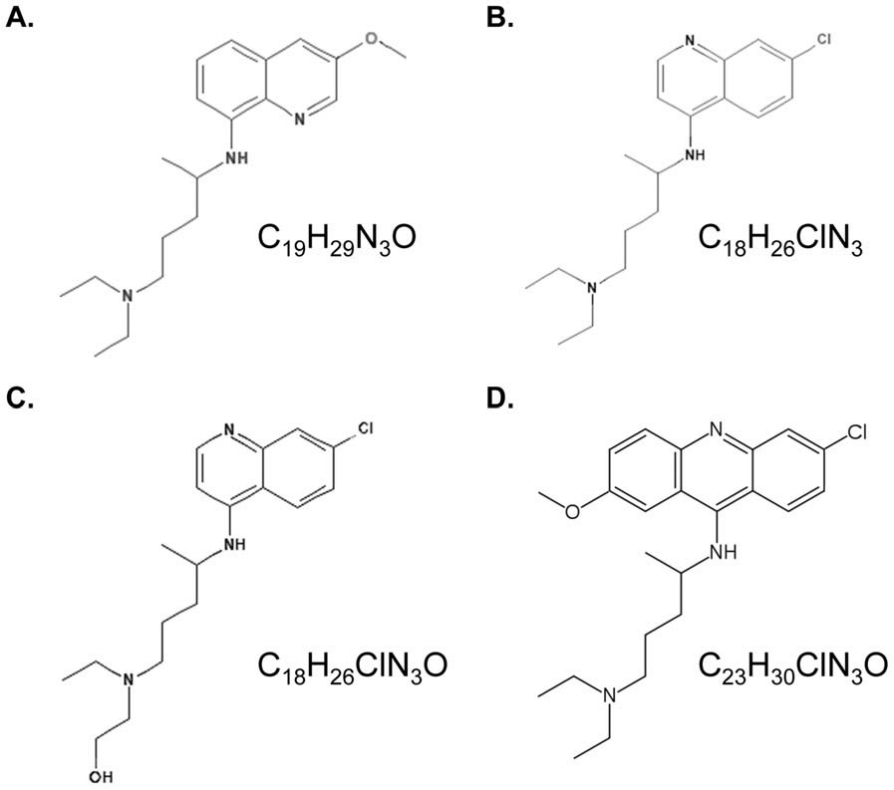
Chemical structures of the NCI compound and structurally similar anti-malarial drugs used in this study. (A) NCI compound NSC56612, (B) chloroquine, (C) hydroxychloroquine, and (D) quinacrine.

Of the 50 candidate compounds from VLS, we were able to procure 32 from the NCI for experimental testing. Screening of the 32 compounds was performed using the Tango assay that tests the inhibition of the CXCL12-mediated recruitment of β-arrestin to the carboxy terminus of CXCR4. This assay identified one hit compound that suppressed β-arrestin recruitment and the chemical structures of this compound NSC56612 is shown in Figure 2. We further performed a gamut of direct ligand binding, secondary messenger calcium flux, and downstream chemotaxis assays mediated by the CXCR4/CXCL12 axis to verify that these compounds are directly targeting CXCR4. NSC56612 was used as a template to identify compounds with similar chemical structures. This lead to identification of three other compounds that are anti-malarial agents: chloroquine, hydroxychloroquine, and quinacrine (Figure 2).

**Figure 3:**
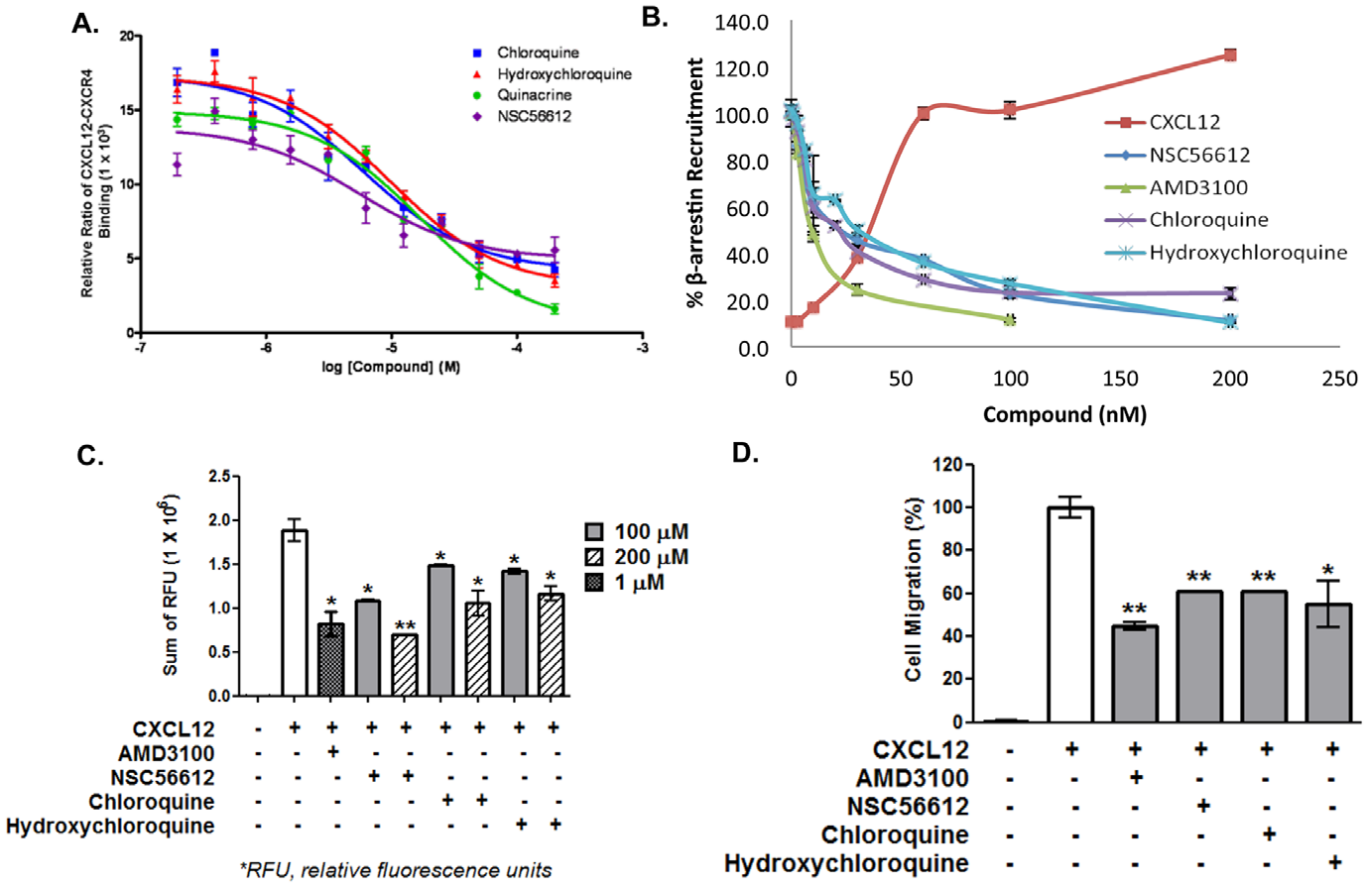
NSC56612, chloroquine, and hydroxychloroquine inhibit CXCL12-mediated activity. (A) Concentration dependent inhibition of fluorescently labeled CXCL12 (60 nM) binding to SNAP-tagged CXCR4 receptor in HEK293 cells by NSC56612, chloroquine, and hydroxychloroquine. The half maximal effective concentrations (EC_50_), the values at which maximum CXCL12-CXCR4 binding is inhibited by 50%, for AMD3100, NSC56612, chloroquine, and hydroxychloroquine are 60.0 nM, 5.5 µM, 6.1 µM, and 9.8 µM, respectively. These curves are representative data from 3 experiments performed in duplicate. (B) Dose-dependent inhibition of CXCL12-induced β-arrestin-2-mediated beta-lactamase activity in engineered Tango assay by pretreatment with AMD 3100, NSC56612, chloroquine, and hydroxychloroquine. Emission data at 460/530 was defined as the response ratio. These curves are representative data from 3 experiments performed in duplicate. (C) Dose-dependent inhibition of CXCL12-induced intracellular calcium flux by AMD 3100, NSC56612, chloroquine, hydroxychloroquine. The CXCR4-mediated calcium influx was measured at 1 µM of AMD3100 and two different concentrations (100 µM and 200 µM) of the three compounds in Molt-4 cells. AMD 3100, NSC56612, chloroquine, and hydroxychloroquine showed 50 to 60% inhibition of CXCL12-induced calcium influx. These curves are representative data from 3 experiments performed in duplicate. (D) Inhibition of CXCL12-induced Jurkat cell migration in by AMD3100 (100 nM), NSC56612 (100 µM), chloroquine (100 µM), and hydroxychloroquine (100 µM). All four compounds showed 40% to 50% reduction in CXCL12-induced cell migration. The figure shows data from 4 experiments performed in duplicate. Error bars represent ± one SD. Student t-test: *<0.05 and **<0.01.

Figure 3A shows the concentration dependence curve of the direct inhibition of fluorescently labeled CXCL12 binding by NSC56612, chloroquine, hydroxychloroquine, and quinacrine. Competitive binding experiments showed that these compounds directly inhibit binding of CXCL12 to CXCR4 with low micro-molar affinity. The assay wherein β-arrestin-2 recruitment mediated by CXCL12 is assessed also showed inhibition by these three compounds with low micro-molar efficacy (Figure 3B). Figure 3B also shows inhibition by AMD3100, a CXCR4 antagonist.

**Figure 4:**
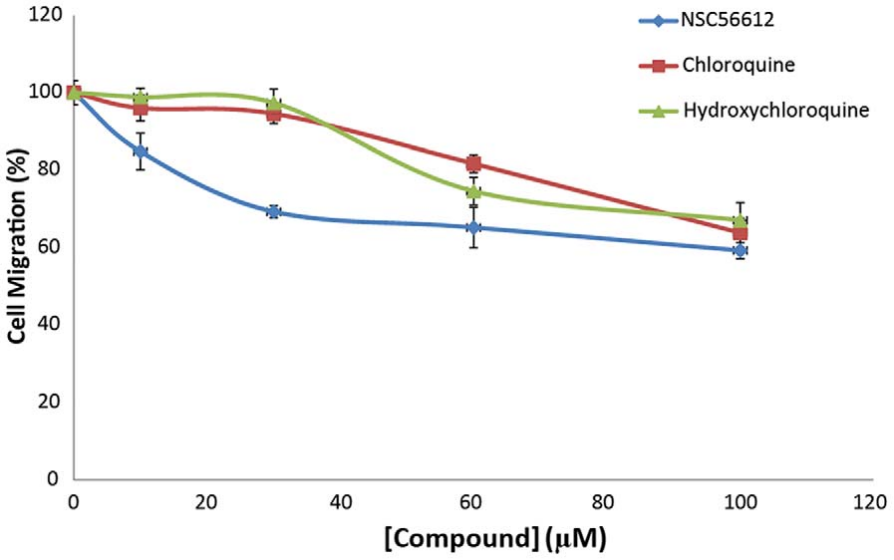
NSC56612, chloroquine, and hydroxychloroquine inhibit CXCL12-induced migration in a dose-dependent manner. Cells were plated in culture plates fitted with 8-uM pore membranes to create upper and lower cell culture chambers. Cells were plated in the upper chamber and CXCL12 (30 nM) alone or with the addition of NSC56612, chloroquine, or hydroxychloroquine was placed in the bottom chamber. The number of cells that migrated through the membrane in 5 hours was counted. Data shown is from 4 experiments performed in duplicate. Relative changes in cell migration are depicted with the control condition serving as 100% migration. Error bars represent ± one SD.

Calcium flux, a secondary messenger to CXCL12-mediated activation of CXCR4, measures the activation of CXCR4. The CXCL12-mediated calcium flux was measured at two different concentrations of the four compounds, namely 100 µM and 200 µM. As seen in Figure 3C, NSC56612, chloroquine, and hydroxychloroquine showed 50% to 60% inhibition of CXCL12-induced calcium flux, while quinacrine showed less than 10% inhibition (data not shown). We measured the level of inhibition by these compounds to chemotaxis, a downstream effect in CXCR4-expressing cells. Figure 3D shows the effect of the compounds in chemotaxis assays wherein the inhibition of CXCL12-induced cell migration is measured. To assess the concentration of compounds necessary for chemotaxis inhibition, we performed a dose-dependent inhibition of chemotaxis of the lead compound NSC56612 as shown in Figure 4. All four compounds showed 40% to 50% reduction in CXCL12-induced cell migration. Quinacrine showed substantial efficacy towards inhibiting chemotaxis (data not shown), while it had little or no effect on the calcium flux assay. Since quinacrine did not suppress CXCL12-mediated calcium flux, it was omitted from further analysis in pancreatic cancer cell lines. The results of these assays show that chloroquine and hydroxychloroquine directly inhibit CXCR4 signaling.

### Chloroquine and Hydroxychloroquine Inhibit CXCL12-Mediated ERK Phosphorylation

**Figure 5:**
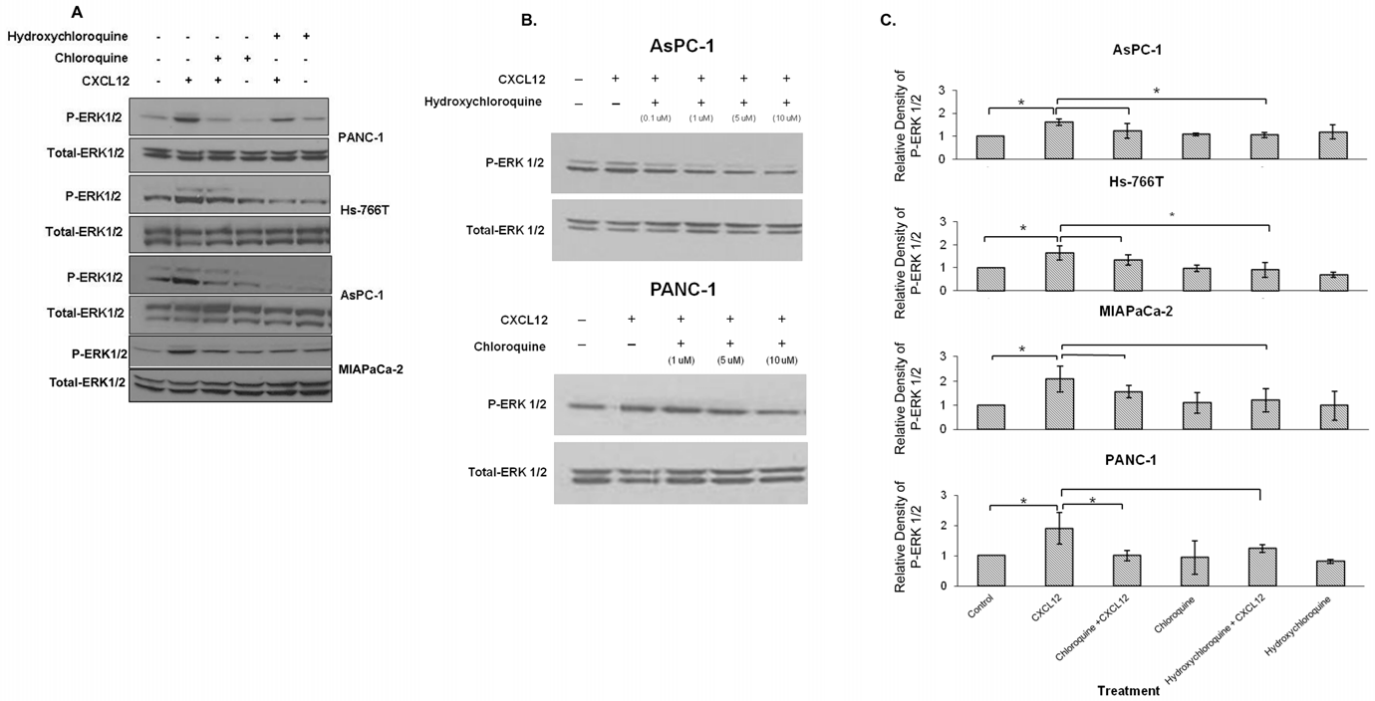
Chloroquine and hydroxhychloroquine inhibit CXCL12-mediated ERK phosphorylation. (A) Immunostaining for phospho-ERK was performed for PANC-1, Hs-766T, AsPC-1, and MIAPaCa-2 cell lysates. Cells were pretreated with chloroquine or hydroxychloroquine (0.1 µM) for 30 minutes after which cells were exposed to CXCL12 (200 ng/ml) for 20 minutes. An antibody to total ERK1/2 was used as a loading control. The CXCL12-mediated increases in phospho-ERK were effectively abrogated with either chloroquine or hydroxychloroquine. (B) Pretreatment of PANC-1 and AsPC-1 cells with chloroquine and hydroxychloroquine (0.1–10 µM) for 5 minutes followed by exposure to CXCL12 (200 ng/ml) demonstrate dose-dependent effects of drug treatment on CXCL12-mediated ERK phosphorylation. (C) Western blots were scanned and quantified using the AlphaImager Tm3400 (Alpha Innotech). Fold changes for phospho-ERK compared to untreated controls were calculated as relative expression, which was normalized to protein band intensities of total ERK. The data shows the mean of triplicate experiments, with *p*-values<0.05 considered statistically significant.

In a previous investigation, we discovered that CXCL12 induced an increase in ERK phosphorylation [21]. Although exposure to CXCL12 also activated the PI-3K/AKT pathway, the degree to which AKT phosphorylation was altered was much lower than ERK phosphorylation. Therefore, we focused our investigation in this study on ERK activation. First, we verified that CXCL12 induces an increase in phospho-ERK in pancreatic cancer cell lines (Figure 5A). Then, we demonstrated that chloroquine and hydroxychloroquine exert dose-dependent inhibitory effects on CXCL12-mediated ERK phosphorylation in PANC-1 and AsPC-1 cells (Figure 5B). Finally, we show the quantitative analysis of the inhibition in phospho-ERK in Figure 5C.

### Chloroquine and Hydroxychloroquine Trigger Apoptosis and Inhibit CXCL12-Mediated Proliferation and Anti-Apoptosis

**Figure 6:**
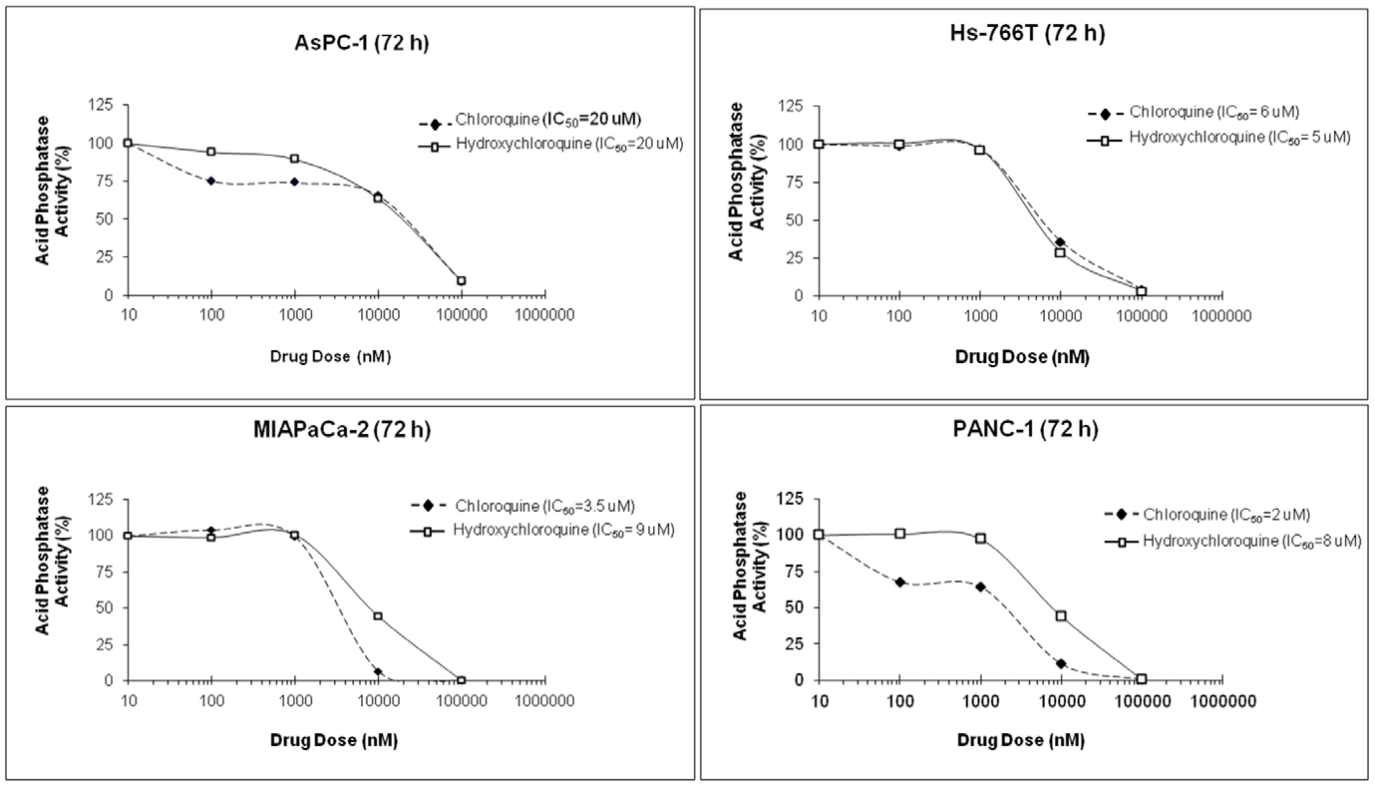
Kill curves for chloroquine and hydroxychloroquine treatment of pancreatic cancer cells. AsPC-1, Hs-766T, MIAPaCa-2, and PANC-1 cells were treated with a dose range of chloroquine or hydroxychloroquine (0.01–10 µM) at 72 hours to determine the half maximal inhibitory concentration (IC50 values) of these compounds. AsPC-1 and Hs-766T cells had similar IC50 values for chloroquine and hydroxychloroquine, whereas MIAPaCa-2 and PANC-1 cells had higher IC50 values for hydroxychloroquine than chloroquine.

**Figure 7:**
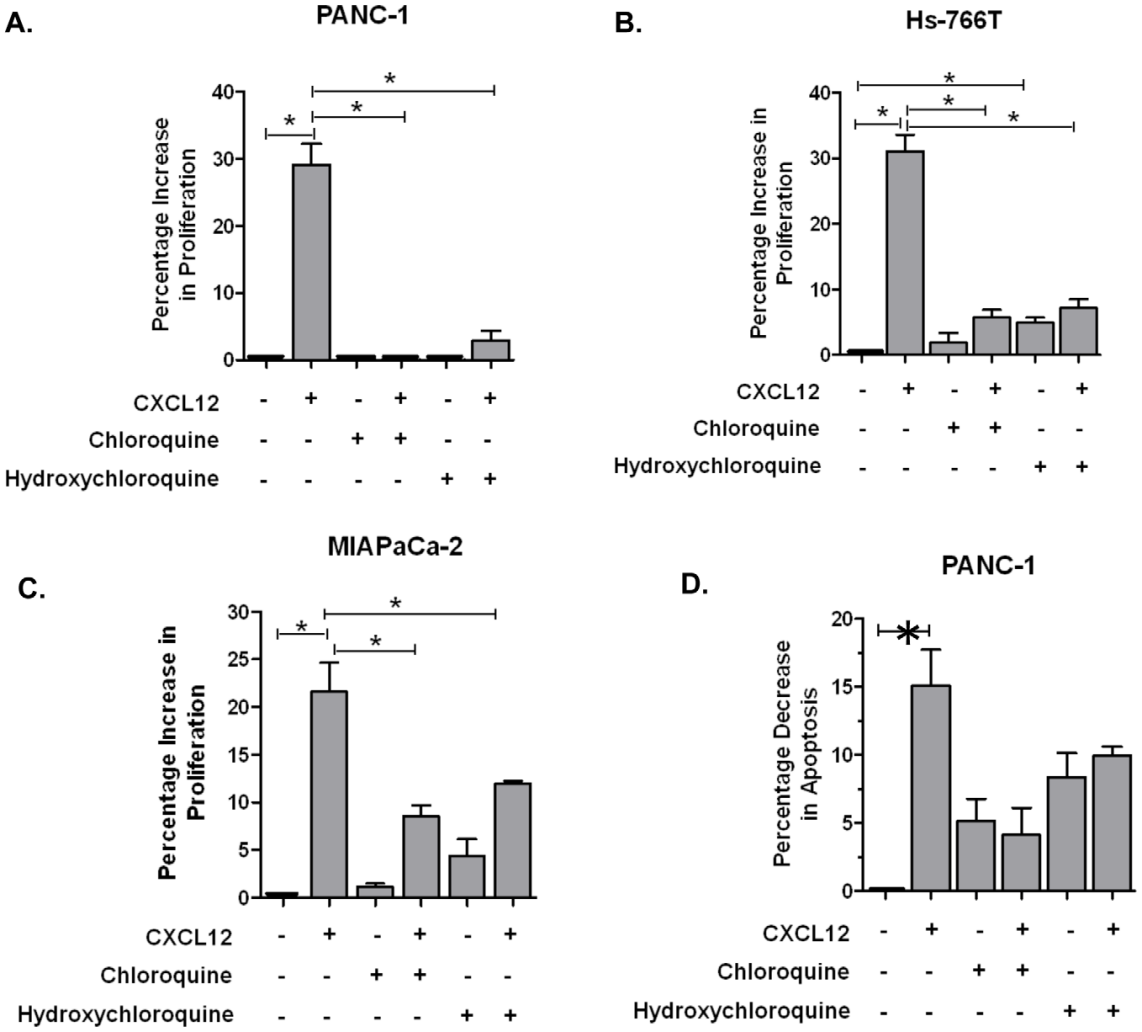
Chloroquine and hydroxychloroquine decrease CXCL12-mediated proliferation in pancreatic cancer cell lines. (A) PANC-1, (B) Hs-766T, and (C) MIAPaCa-2 cells were pretreated with chloroquine or hydroxychloroquine (0.1 µM) for 30 minutes after which cells were exposed to CXCL12 (200 ng/ml) for 72 hours. (D) Pretreatment with chloroquine and hydroxychloroquine also triggered apoptosis and decreased CXCL12-mediated apoptosis in PANC-1 cells. The data show the mean of triplicate experiments. *P*-values<0.05 were considered statistically significant.

Chloroquine and hydroxychloroquine cytotoxicity in pancreatic cancer cell lines was assessed and the IC50s were determined (Figure 6). Using these values, we evaluated CXCR4 signaling in a cell proliferation assay. We previously observed CXCR4-mediated increases in cell proliferation in pancreatic cancer cells [21]. Therefore, chloroquine and hydroxychloroquine were assessed for antagonism of CXCR4-mediated cell proliferation; and we observed that both agents effectively antagonized CXCR4-mediated cell proliferation in PANC-1, Hs-766T, and MIAPaCa-2 cells (Figure 7). Since increased proliferation was not observed in AsPC-1 cells after exposure to CXCL12, these cells were not assessed with chloroquine and hydroxychloroquine in this assay. Our results are consistent with published reports which show that not all pancreatic cancer cell lines respond to CXCL12 with increased proliferation [2]; the mechanism responsible for this has not yet been elucidated. Both chloroquine and hydroxychloroquine showed reduction in phospho-ERK in the presence of CXCL12, with the total ERK concentration being unaffected. These results show that chloroquine and hydroxychloroquine specifically inhibit CXCR4-mediated signaling to suppress cell proliferation in pancreatic cancer cells.

Since the mechanism for changes in cell proliferation was not clear, we also assessed apoptosis following exposure to chloroquine and hydroxychloroquine. First, our results suggest that CXCL12 promotes anti-apoptosis in pancreatic cancer cells. These results are consistent with previously published reports for the CXCL12/CXCR4 axis [22], [23]. Second, our results indicate that chloroquine and hydroxychloroquine abrogate CXCL12-mediated anti-apoptosis (Figure 7D), wherein pretreatment with chloroquine or hydroxychloroquine increased apoptosis in PANC-1 cells.

## Discussion

Using a cross-disciplinary approach starting with computational modeling of the CXCR4 receptor structure to *in vitro* analysis of CXCR4 signaling, our study has determined that chloroquine and hydroxychloroquine act as novel CXCR4 inhibitors in pancreatic cancer cells. We have demonstrated that these clinically safe and effective anti-malarial agents specifically inhibit binding of CXCL12 to CXCR4 and inhibit CXCL12-CXCR4 downstream effector pathways that mediate calcium flux, recruitment of ß-arrestin-2 and cell migration. In pancreatic cancer cell lines we determined that chloroquine and hydroxychloroquine block CXCL12-mediated signaling through the ERK pathway with downstream effects on both apoptosis and cell proliferation. Since CXCR4 appears to have an important role in the pathogenesis and progression of pancreatic cancer [1], [2], [3], our work has important clinical implications in the identification of a novel therapeutic use for these established anti-malarial agents.

**Figure 8:**
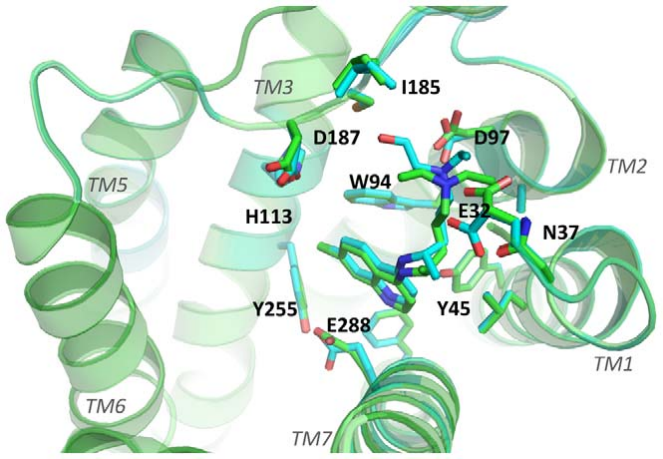
Top view of the predicted ligand binding site in the crystal structure of human CXCR4. Green helices, TM1, 2, 3, 5, 6, and 7 are shown. Upon binding, both chloroquine and hydroxychloroquine interact favorably with the indicated residues (E32, N37, Y45, W94, D97, H113, I185, D187, Y255, E288) shown in sticks. These residues are within 5 Å of the bound compound. However, the amino acid residues at the CXCR4 binding site are oriented slightly different depending on whether chloroquine or hydroxychloroquine bind. The relative orientations of the CXCR4 amino acid residues that interact with chloroquine and hydroxychloroquine are shown as green and cyan sticks, respectively.

Our initial studies required the accurate characterization of the structure of CXCR4. Using computational methods previously developed by us, we predicted the three-dimensional structure of CXCR4 and the binding sites of known small molecule antagonists such as cyclam compounds [16], [24], [25]. Our structural predictions were performed prior to the publication of the crystal structure of CXCR4 [17]. Subsequent comparison of the predicted structures to the crystal showed an excellent agreement of the root mean square deviation in coordinates of the ligand of 2.2 Å. These results were encouraging to further our study looking for small molecule CXCR4 antagonists in pancreatic cancer cells. With the predicted conformations of CXCR4, we used established libraries of compounds to identify candidate antagonist hits. Limiting the hits to the top 32 candidates, we performed high-throughput screening assays, which further narrowed our candidate list to 3 compounds. These initial studies led us to NCI compound NSC56612. Exploration of the chemical structure of NSC56612 for similar compounds via public and commercial databases revealed that NSC56612 shared structural homology to chloroquine, hydroxychloroquine, and quinacrine; but only chloroquine and hydroxychloroquine passed all of our screening assays. The disparate efficacy of quinacrine could be secondary to the heterogeneity of downstream effectors (*e.g.*, phospholipase C or G-proteins) for different cellular functions, which may result in variable response between cells. Other studies have previously demonstrated differential efficacy of discrete G protein coupled receptor ligands for different assays [26], [27]. To identify the putative binding sites of these compounds we docked chloroquine and hydroxychloroquine to the crystal structure of CXCR4 (Figure 8) and identified that the tertiary amine groups in these compounds make hydrogen bonds with D97 on TM2 and E32 in the amino terminus of the receptor; and aromatic residues Y45, W94, H113 and Y255 show favorable van der Waals interaction with the aromatic ring system in these compounds.

Since the invasive phenotype associated with CXCR4 is a manifestation of CXCL12-driven signaling pathways, we tested chloroquine and hydroxychloroquine *in vitro*. We chose to evaluate the MAPK signaling pathway, because we have previously demonstrated its role in mediating growth and proliferation in pancreatic intraepithelial neoplasia and pancreatic cancer cells [21]. In our current studies we observed effective inhibition of CXCL12-driven ERK phosphorylation and inhibition of CXCL12-mediated proliferation *in vitro*.

There is clinical evidence for the anti-cancer effects of chloroquine and hydroxychloroquine. In a clinical trial for glioblastoma multiforme, chloroquine was administered along with conventional chemotherapy as adjuvant therapy in patients who underwent surgical resection for glioblastoma multiforme. The patients who received chloroquine experience improved survival compared to patients who received chemotherapy alone [28]. Although they did not identify CXCR4 as a potential target, Rubin *et al.*, had previously shown that CXCR4 antagonism inhibited glioblastoma multiforme growth, suggesting that CXCR4 was an appropriate target for glioblastoma therapy [29]. Additionally, a clinical trial for metastatic colorectal cancer has incorporated hydroxychloroquine along with standard cytotoxic chemotherapy [30]. Our group and others have examined CXCR4 expression in colorectal cancer and observed a potential role for CXCR4 in colorectal cancer progression [4], [31], [32]. Our study demonstrates that chloroquine and hydroxychloroquine are antagonists to CXCR4 and thus provides a molecular basis for using chloroquine in patients with metastatic colorectal cancer. Since this clinical trial is ongoing, the results have yet to mature. Chloroquine has also been recently tested *in vivo* in a murine pancreatic cancer model demonstrating tumoricidal effects with improved survival [33]. However, these investigators used chloroquine without an understanding of its effects on CXCR4.

Although chloroquine and hydroxychloroquine have been recently used for anti-cancer applications, they were originally formulated as anti-malarial agents. These drugs were formulated because Atabrine, the first synthetic anti-malarial compound, had undesirable side-effects of staining the skin and eyes [34]. Both chloroquine and hydroxychloroquine are weak bases that target blood cells that are infected by malaria [35]. These drugs work by preferentially diffusing into the parasite's vacuole where hemoglobin is broken down [36]. In the acidic vacuole, they become protonated and trapped [36]. Within the vacuole, they inhibit the breakdown of heme, the byproduct of parasitic degradation of hemoglobin [36]. The accumulation of heme becomes toxic and leads to cell lysis and death of the parasite [36]. Aside from these anti-malarial indications, the mechanism for the anti-neoplastic effects of chloroquine and hydroxychloroquine have been examined [37]. Chloroquine appears to inhibit autophagy and induce p53-dependent apoptosis [38]; and may also enhance the effects of chemotherapy or radiation therapy [39].

In conclusion, using a cross-disciplinary approach we identified novel CXCR4 antagonists and have shown that chloroquine and hydroxychloroquine inhibit CXCL12-CXCR4 signaling. Given that effective antagonists to CXCR4 are lacking and that novel therapies have yet to improve survival beyond 1-year for patients with metastatic pancreatic cancer [25], [40], [41], our results have important clinical implications. Our study results provide a scientific basis for using chloroquine or hydroxychloroquine in a pancreatic cancer trial; and since the safety profiles are well established for these drugs, a clinical trial can be expeditiously implemented for patients with pancreatic cancer.

## Materials and Methods

### Prediction of the CXCR4 Structure

MembStruk4.3, the *ab initio* structure prediction method, was used to generate an ensemble of wild-type human CXCR4 structures [14], [15]. The trans-membrane (TM) regions of CXCR4 were predicted using the Tm2ndS method with multiple sequence alignment of human, rat, and mouse CXC and CC family of chemokine receptors (Table 1) [14]. We optimized the relative rotation and translation of the seven TM helices and the helical kinks beginning from an assembled bundle of canonical helices built from the TM predictions. Canonical right-handed α-helices were built for each helix and their helical axes were oriented in space according to the 7.5 Å low-resolution electron density map of frog rhodopsin [42].

**Table 1:** Predicted transmembrane regions of the human CXCR4 receptor.

NT	1 MEGISIYTSDNYTEEMGSGDYDSMKEPCFREENANF 36 (36)
TM 1	37 NKIFLPTIYSIIFLTGIVGNGLVILVMG 64 (28)
LP 1	65 YQKKLRSMTD 74 (10)
TM 2	75 KYRLHLSVADLLFVITLPFWAVDA 98 (24)
LP 2	99 VANWYFG 105 (7)
TM 3	106 NFLCKAVHVIYTVNLYSSVLILAFISLDRYL 136 (31)
LP 3	137 AIVHATNSQRPRKLLA 152 (16)
TM 4	153 EKVVYVGVWIPALLLTIPDFIFANVSE 179 (27)
LP 4	180 ADDRYICDRFYPNDLWVVV 198 (19)
TM 5	199 FQFQHIMVGLILPGIVILSCYCIIISK 225 (27)
LP 5	226 LSHSKGHQKRKAL 238 (13)
TM 6	239 KTTVILILAFFACWLPYYIGISIDSFIL 266 (28)
LP 6	267 LEIIKQGCEFENTVHKW 283 (17)
TM 7	284 ISITEALAFFHCCLNPILYAFLG 306 (23)
CT	307 AKFKTSAQHALTSVSRGSSLKILSKGKRGGHSSVSTESESSSFHSS 352 (46)

Amino-terminal region (NT), Transmembrane region (TM), Loop region (LP), Carboxy-terminal region (CT).

This 7.5 Å electron density map provided the positions and relative orientations of the helical axes that served to optimize the helical bundle. The relative translational orientations of the 7 helices were optimized by aligning the hydrophobic maximum determined for each helix to a plane. The rotational orientation was optimized using a combination of hydrophobic moments and molecular dynamic techniques. Data from the crystal structure of CXCR4 [17], which was only recently characterized, was not used for these predictions.

We derived an ensemble of low energy TM barrel conformations for CXCR4 and its constitutively active mutants [43]. The receptor conformations were selected to have the maximum number of inter-helical hydrogen bonds and the highest total energy of the protein conformation in a lipid bilayer. Three potential low energy conformations were selected for CXCR4 that had different orientations of TM3, TM5, and TM7. TM5 had 3 different conformations: 2 had different orientations of the residue Y219^5.58^, which correspond to the 2 different orientations of Y223^5.58^ in rhodopsin and opsin crystal structures [44], [45]. TM7 had 2 different helical orientations, where the position of E288^7.39^ was different.

### Docking of AMD3100 and Virtual Ligand Screening

The predicted binding site of the mono and bicyclam derivatives of AMD3100 on CXCR4 was validated using established site-directed mutagenesis data [19], [20]. This predicted binding site was then used to perform virtual ligand screening (VLS) to identify new antagonist hits for CXCR4. The National Cancer Institute's (NCI) Open Chemical Repository Collection [46], which is composed of approximately 300,000 compounds, was queried using the Maestro LigPrep module (Schrödinger; San Diego, CA). Multiple conformations of candidate ligands were then docked into the predicted binding site of CXCR4 using Glide SP (Schrödinger) scaling the van der Waals radii to 0.5 and partial charge cutoff to 0.15. The combined Coulombic and van der Waals energy cutoffs were then raised to 100 kcal/mol. The charged molecules were eliminated and the neutral molecules that were considered better candidates were selected for further investigation. The docked ligand conformations for neutral molecules were then filtered based on the buried surface area, wherein ligands that were >80% buried and based on their distances to the acidic residues D171, D262, and E288 were selected. These 3 residues have been shown to be important in binding CXCR4 antagonists [19], [20].

Using the Prime module in Maestro, the side-chains within 5 Å radius of the ligand were reassigned. Following the side-chain reassignment, the binding energies (BE) of each docked conformation was calculated using BE = PE (ligand in fixed protein) - PE (ligand in solvation), where PE (ligand in fixed protein) is the potential energy of the ligand calculated with the protein atoms fixed and PE (ligand in solvation) is the potential energy of the ligand calculated with the Surface Generalized Born continuum solvation method [47]. The top 200 conformations of each set were then visually inspected to maximize favorable receptor interactions. We then selected the top 50 compounds from each CXCR4 receptor conformation for subsequent testing.

### Fluoresence Labeled Competitive Ligand Binding Assay

Competitive binding studies to evaluate candidate CXCR4 antagonists were performed using the chemokine CXCR4 receptor ligand binding assay kit according to manufacturer's instructions (Tag-lite, Cisbio; Bedford, MA) [48], [49]. Briefly, HEK-293 cells were incubated at room temperature for 2 hours with the fluorescent-labeled CXCL12 ligand with or without the CXCR4 antagonists. After incubation, the cells were excited at 620 nm and recorded at dual-emissions (620 nm and 665 nm) using PHERAstar (BMG LABTECH Inc.; Cary, NC). The relative ratios of CXCL12-CXCR4 binding were obtained by dividing the acceptor signal (665 nm) by the donor signal (620 nm) and multiplying this value by 10,000.

### CXCR4 Recruitment of β-arrestin

Activation of the CXCL12-CXCR4 axis results in the recruitment of β-arrestin-2 to the carboxy terminus of CXCR4 [50]. To verify the inhibition of β-arrestin-2 recruitment by candidate CXCR4 antagonists, we used a commercial CXCR4 assay (Tango; Invitrogen, Carlsbad, CA) [51], [52]. Briefly, the engineered cells (3×10^4^) were seeded in 96-well plates and incubated overnight. DMSO or antagonists were added to the cells for 30 minutes. Then CXCL12 (60 nM) was added and cells were incubated for 5 hours. Then, LiveBLAzer™-FRET B/G substrate mixture (24 µl) (Invitrogen) was added and incubated in the dark at room temperature for 2 hours. Plates were then read on a Synergy microplate reader (BioTek; Winooski, VT) with excitation at 409 nm and emission at 460 nm and 530 nm. Background fluorescence values for each emission wavelength were obtained from cell-free wells containing assay medium and LiveBLAzer™-FRET B/G substrate and subtracted from the fluorescence values of the test wells. The background-corrected fluorescence emission values at 460 nm were divided by the 530 nm value to obtain a 460 nm/530 nm ratio. The percentage of β-arrestin recruitment of the sample was then calculated by dividing the 460 nm/530 nm ratio of the testing well by the 460 nm/530 nm ratio of CXCL12 control well.

### Calcium Flux Assay

A calcium flux assay was performed to assess anti-CXCR4 compounds using a Fluo-4 Direct calcium assay (Invitrogen) using Molt-4 cells. These cells (8×10^5^) were seeded in 96-well plates with 2× Fluo-4 Direct calcium reagents. After incubation, candidate antagonists were added to the wells and incubated at room temperature for 1 hour. Changes in intracellular calcium concentration upon addition of CXCL12 (50 nM) were monitored by fluorescence excited at 494 nm and emitted at 516 nm using a Synergy reader (BioTek). The sum of relative fluorescence units (RFU) was calculated as the area under the equation derived from continuous values of emission at 516 nm over a period of 90 seconds. The data represents three experiments performed in duplicate.

### Migration Assay

A migration assay to assess antagonists to CXCL12-mediated chemotaxis was performed in 24-well cell culture plates with 8-µm pore polycarbonate membranes (Millipore; Billerica, MA) using the Jurkat cells. Briefly, cells (5×10^6^) were placed into the upper chamber and CXCL12 (30 nM) in the lower chamber. Cell migration was measured after incubation at 37°C for 5 hours. For inhibition of migration, candidate antagonists and CXCL12 were placed into the lower chamber. Migrating cells were harvested and counted by hemacytometer. The percentage cell migration was quantified as the ratio of the total number of cells in all of the wells to the cells in the CXCL12 control wells (30 nM CXCL12), scaled to the CXCL12 control well as 100% cell migration. These experiments were performed in triplicate.

### Cell Culture and Reagents for CXCR4 Signaling Assays

The established human pancreatic cancer cell lines PANC-1, Hs-766T, AsPC-1, and MIAPaCa-2 were obtained from the American Type Culture Collection (Manassas, VA) less than 5 years ago. All cells used for the experiments in this study were from cryopreserved stores frozen in liquid nitrogen at the time that the cell lines were commercially obtained. Polymerase chain reaction (PCR) and direct sequencing were performed for K-*ras* and *p53* mutations to verify the genotype of the cells. Cells were cultured in recommended media and maintained at 37°C and 5% CO_2_. Chloroquine and hydroxychloroquine were purchased from MP Biomedical (Solon, OH).

### Antagonism of ERK Phosphorylation

We have previously demonstrated CXCL12-driven increases in ERK phosphorylation in pancreatic cancer cells [21]. CXCL12-driven changes in ERK phosphorylation following pre-treatment with chloroquine and hydroxychloroquine were assessed by Western blot assay as described [21]. 50 µg of cell lysates were resolved on a 12% sodium dodecyl sulfate (SDS)-polyacrylamide gel and transferred to a polyvinylidene fluoride (PVDF) membrane. Membranes were blocked in blocking buffer for 1 hour and then probed with primary antibodies at 4°C overnight. After probing with horseradish peroxidase conjugated secondary antibodies, presence of specific proteins on the Western blot was detected using ECL reagent (Thermo Scientific; Rockford, IL). Relative immunoblot band intensities were quantified using densitometry (Alpha Innotech; Santa Clara, CA).

### Measurement of Cytotoxicity

Chloroquine and hydroxychloroquine (0.1–100 µM) were tested for cytotoxic activity in PANC-1, Hs-766T, ASPC-1, and MIAPaCa-2 cells. Compounds were incubated with cells for 72 hours and cytotoxicity was measured via cellular acid phosphatase activity. In brief, cells were seeded in 96-well plates. After 72 hours, media was removed. Para-Nitrophenyl phosphate (pNPP) buffer (0.1 M sodium acetate [pH 5.5], 0.1% Triton x100, 10 mM pNPP [N4645, Sigma], phosphate-buffered solution) was added to each well and cells were incubated at 37°C for 2 hours. After 1 N NaOH was added to each well, plates were then read using the SpectraMax M2 microplate reader at 405 nm (Molecular Devices, Sunnyvale, CA). At least 3 independent assays were performed for each cell line. The minimal inhibitory concentration (IC_50_) at which ½ the cells were still viable was determined for each compound in the 3 cell lines.

### Cell Proliferation Assay

We have previously demonstrated CXCL12-driven increases in pancreatic cancer cell proliferation [21]. CXCL12-driven cell proliferation was measured following pre-treatment with chloroquine and hydroxychloroquine. CellTiter-Glo assay (Promega) was used to detect cell proliferation as described [21]. In brief, cells were plated in 96-well plates at a density of 5×10^3^ cells per well. Cells were exposed to either chloroquine (0.1 µM) or hydroxychloroquine (0.1 µM) for 30 minutes and then exposed to CXCL12 (200 ng/ml) for 72 hours. For detection of the luminescent signal, CellTiter-Glo reagent was added and the plates were incubated and measured on a luminometer (Perkin-Elmer, Shelton, CT). The level of proliferation of untreated cells (*i.e.*, control cells), was normalized to zero. The cell proliferation of the treatment arms were then compared against these control cells. At least 3 independent cell proliferation assays were performed for each cell line.

### Apoptosis Assay

The effects on CXCL12-driven anti-apoptosis were assessed following exposure to chloroquine and hydroxychloroquine. Pancreatic cancer cells (3×10^5^) were maintained in 6-well plates, serum-starved for 24 hours, and then incubated with chloroquine (0.1 µM) or hydroxychloroquine (0.1 µM) for 30 minutes. Cells were then exposed to CXCL12 (200 ng/ml) for 48 hours. Apoptotic cells were assessed by an Annexin V assay according to the manufacturer's protocol (Invitrogen). In brief, 1×10^5^ cells were washed with cold PBS and re-suspended in 1× Annexin-binding buffer to a final volume of 100 µl with Alexa Fluor 488, annexin V, and propidium iodide. The mixture suspension was gently vortexed and incubated for 15 minutes in the dark at room temperature. Cells were then analyzed by flow cytometric assay.

## References

[1] Koshiba T, Hostonai R, Miyamoto Y, Ida J, Tsuji S (2000). Expression of stromal cell-derived factor 1 and CXCR4 ligand receptor system in pancreatic cancer: a possible role for tumor progression.. Cancer Res.

[2] Marchesi F, Monti P, Leone B, Zerbi A, Vecchi A (2004). Increased survival, proliferation, and migration in metastatic human pancreatic tumor cells expressing functional CXCR4.. Cancer Res.

[3] Saur D, Seidler B, Schneider G, Algül H, Beck R (2005). CXCR4 expression increases liver and lung metastasis in a mouse model of pancreatic cancer.. Gastroenterology.

[4] Kim J, Mori T, Chen S, Amersi F, Martinez S (2006). Chemokine receptor CXCR4 expression in patients with melanoma and colorectal cancer liver metastases and the association with disease outcome.. Ann Surg.

[5] Hatse S, Princen K, Bridger G, De Clercq E, Schols D (2002). Chemokine receptor inhibition by AMD3100 is strictly confined to CXCR4.. FEBS Lett.

[6] De Clercq E (2005). Potential clinical applications of the CXCR4 antagonist bicyclam AMD3100.. Mini Rev Med Chem.

[7] Singh S, Srivastava S, Bhardwaj A, Owen L, Singh A (2010). CXCL12-CXCR4 signalling axis confers gemcitabine resistance to pancreatic cancer cells: a novel target for therapy.. Br J Cancer.

[8] Ratajczak M, Zuba-Surma E, Kucia M, Reca R, Wojakowski W (2006). The pleiotropic effects of the SDF-1-CXCR4 axis in organogenesis, regeneration and tumorigenesis.. Leukemia.

[9] Feng Y, Broder C, Kennedy P, Berger E (1996). HIV-1 entry cofactor: functional cDNA cloning of a seven-transmembrane, G protein-coupled receptor.. Science.

[10] Endres M, Clapham P, Marsh M, Ahuja M, Turner J (1996). CD4-independent infection by HIV-2 is mediated by fusin/CXCR4.. Cell.

[11] Hendrix C, Collier A, Lederman M, Schols D, Pollard R (2004). Safety, pharmacokinetics, and antiviral activity of AMD3100, a selective CXCR4 receptor inhibitor, in HIV-1 infection.. J Acquir Immune Defic Syndr.

[12] Kalatskaya I, Berchiche Y, Gravel S, Limberg B, Rosenbaum J (2009). AMD3100 is a CXCR7 ligand with allosteric agonist properties.. Mol Pharmacol.

[13] Gravel S, Malouf C, Boulais P, Berchiche Y, Oishi S (2010). The peptidomimetic CXCR4 antagonist TC14012 recruits beta-arrestin to CXCR7: roles of receptor domains.. J Biol Chem.

[14] Trabanino R, Hall S, Vaidehi N, Floriano W, Kam V (2004). First principles predictions of the structure and function of G-protein-coupled receptors: validation for bovine rhodopsin.. Biophys J.

[15] Heo J, Han S, Vaidehi N, Wendel J, Kekenes-Huskey P (2007). Prediction of the 3D structure of FMRF-amide neuropeptides bound to the mouse MrgC11 GPCR and experimental validation.. Chembiochem.

[16] Lam A, Bhattacharya S, Patel K, Hall S, Mao A (2010). Importance of receptor flexibility in binding of cyclam compounds to the chemokine receptor CXCR4.. J Chem Inf Model.

[17] Wu B, Chien E, Mol C, Fenalti G, Liu W (2010). Structures of the CXCR4 chemokine GPCR with small-molecule and cyclic peptide antagonists.. Science.

[18] Kufareva I, Rueda M, Katritch V, Stevens R, 2010 PoGD (2011). Status of GPCR modeling and docking as reflected by community wide GPCR Dock 2010 assessment.. Structure.

[19] Rosenkilde M, Gerlach L, Hatse S, Skerlj R, Schols D (2007). Molecular mechanism of action of monocyclam versus bicyclam non-peptide antagonists in the CXCR4 chemokine receptor.. J Biol Chem.

[20] Wong R, Bodart V, Metz M, Labrecque J, Bridger G (2008). Comparison of the potential multiple binding modes of bicyclam, monocylam, and noncyclam small-molecule CXC chemokine receptor 4 inhibitors.. Mol Pharmacol.

[21] Shen X, Artinyan A, Jackson D, Thomas R, Lowy A (2010). Chemokine receptor CXCR4 enhances proliferation in pancreatic cancer cells through AKT and ERK dependent pathways.. Pancreas.

[22] Denizot M, Varbanov M, Espert L, Robert-Hebmann V, Sagnier S (2008). HIV-1 gp41 fusogenic function triggers autophagy in uninfected cells.. Autophagy.

[23] Lipinski M, Hoffman G, Ng A, Zhou W, Py B (2010). A genome-wide siRNA screen reveals multiple mTORC1 independent signaling pathways regulating autophagy under normal nutritional conditions.. Dev Cell.

[24] Bhattacharya S, Vaidehi N (2010). Computational mapping of the conformational transitions in agonist selective pathways of a G-protein coupled receptor.. J Am Chem Soc.

[25] Philip P, Benedetti J, Fenoglio-Preiser C, Zalupski M, Lenz H (2007). Phase III study of gemcitabine [G] plus cetuximab [C] versus gemcitabine in patients [pts] with locally advanced or metastatic pancreatic adenocarcinoma [PC]: SWOG S0205 study.. J Clin Oncol.

[26] Kilts J, Connery H, Arrington E, Lewis M, Lawler C (2002). Functional selectivity of dopamine receptor agonists. II. Actions of dihydrexidine in D2L receptor-transfected MN9D cells and pituitary lactotrophs.. J Pharmacol Exp Ther.

[27] Whalen E, Rajagopal S, Lefkowitz R (2011). Therapeutic potential of beta-arrestin- and G protein-biased agonists.. Trends Mol Med.

[28] Sotelo J, Briceño E, López-González M (2006). Adding chloroquine to conventional treatment for glioblastoma multiforme: a randomized, double-blind, placebo-controlled trial.. Ann Intern Med.

[29] Rubin J, Kung A, Klein R, Chan J, Sun Y (2003). A small molecule antagonist of CXCR4 inhibits intracranial growth of primary brain tumors.. Proc Natl Acad Sci USA.

[30] Moss RA (2009). Autophagy and Anti-Angiogenesis in Metastatic Colorectal Carcinoma: A Phase II Trial of Hydroxychloroquine to Augment Effectiveness of XELOX-Bevacizumab. A Study of the Cancer Institute of New Jersey Oncology Group.. http://www.clinicaltrials.gov.

[31] Kim J, Takeuchi H, Lam S, Turner R, Wang H (2005). Chemokine receptor CXCR4 expression in colorectal cancer patients increases the risk for recurrence and for poor survival.. J Clin Oncol.

[32] Zeelenberg I, Ruuls-Van Stalle L, Roos E (2003). The chemokine receptor CXCR4 is required for outgrowth of colon carcinoma micrometastases.. Cancer Res.

[33] Yang S, Wang X, Contino G, Liesa M, Sahin E (2011). Pancreatic cancers require autophagy for tumor growth.. Genes Dev.

[34] Coatney R (1963). Pitfalls in a discovery: the chronicle of chloroquine.. Am J Trop Med Hyg.

[35] Warhurst D, Steele J, Adagu I, Craig J, Cullander C (2003). Hydroxychloroquine is much less active than chloroquine against chloroquine-resistant Plasmodium falciparum, in agreement with its physicochemical properties.. J Antimicrob Chemother.

[36] Hempelmann E (2007). Hemozoin biocrystallization in *Plasmodium falciparum* and the antimalarial activity of crystallization inhibitors.. Parasitol Res.

[37] Ben-Zvi I, Kivity S, Langevitz P, Shoenfeld Y (2011). Hydroxychloroquine: from malaria to autoimmunity.. Clin Rev Allergy Immunol.

[38] Maclean K, Dorsey F, Cleveland J, Kastan M (2008). Targeting lysosomal degradation induces p53-dependent cell death and prevents cancer in mouse models of lymphomagenesis.. J Clin Invest.

[39] Sasaki K, Tsuno N, Sunami E, Tsurita G, Kawai K (2010). Chloroquine potentiates the anti-cancer effect of 5-fluorouracil on colon cancer cells.. BMC Cancer.

[40] Di Marco M, Di Cicilia R, Macchini M, Nobili E, Vecchiarelli S (2010). Metastatic pancreatic cancer: is gemcitabine still the best standard treatment? (Review).. Oncol rep.

[41] Moore M, Goldstein D, Hamm J, Figer A, Hecht J (2007). Erlotinib plus gemcitabine compared with gemcitabine alone in patients with advanced pancreatic cancer: a phase III trial of the National Cancer Institute of Canada Clinical Trials Group.. J Clin Oncol.

[42] Schertler G (1998). Structure of rhodopsin.. Eye (Lond).

[43] Wang Z, Broach J, Peiper S (2006). Functional expression of CXCR4 in Saccharomyces cerevisiae in the development of powerful tools for the pharmacological characterization of CXCR4.. Methods Mol Biol.

[44] Li J, Edwards P, Burghammer M, Villa C, Schertler G (2004). Structure of bovine rhodopsin in a trigonal crystal form.. J Mol Biol.

[45] Park J, Scheerer P, Hofmann K, Choe H, Ernst O (2008). Crystal structure of the ligand free G-protein coupled receptor opsin.. Nature.

[46] Ihlenfeldt W, Voigt J, Bienfait B, Oellien F, Nicklaus M (2002). Enhanced CACTVS browser of the Open NCI Database.. J Chem Inf Comput Sci.

[47] Ghosh A, Rapp C, Friesner R (1998). Generalized Born model based on a surface integral formulation.. J Phys Chem B.

[48] Zwier J, Roux T, Cottet M, Durroux T, Douzon S (2010). A fluorescent ligand-binding alternative using Tag-lite® technology.. J Biomol Screen.

[49] Leyris J, Roux T, Trinquet E, Verdié P, Fehrentz J (2011). Homogeneous time-resolved fluorescence-based assay to screen for ligands targeting the growth hormone secretagogue receptor type 1a.. Anal Biochem.

[50] McCormick P, Segarra M, Gasperini P, Gulino A, Tosato G (2009). Impaired recruitment of Grk6 and beta-Arrestin 2 causes delayed internalization and desensitization of a WHIM syndrom-associated CXCR4 mutant receptor.. PLoS One.

[51] Doucette C, Vedvik K, Koepnick E, Bergsma A, Thomson B (2009). Kappa opioid receptor screen with the Tango beta-arrestin recruitment technology and characterization of hits with second-messenger assays.. J Biomol Screen.

[52] Hanson B, Wetter J, Bercher M, Kopp L, Fuerstenau-Sharp M (2009). A homogeneous fluorescent live-cell assay for measuring 7-transmembrane receptor activity and agonist functional selectivity through beta-arrestin recruitment.. J Biomol Screen.

